# Neuro-cognitive mechanisms of conscious and unconscious visual perception: From a plethora of phenomena to general principles

**DOI:** 10.2478/v10053-008-0090-4

**Published:** 2011-12-01

**Authors:** Markus Kiefer, Ulrich Ansorge, John-Dylan Haynes, Fred Hamker, Uwe Mattler, Rolf Verleger, Michael Niedeggen

**Affiliations:** 1 Department of Psychiatry, University of Ulm, Germany; 2 Department of Psychology, University of Vienna, Austria; 3 Bernstein Center for Computational Neuroscience, Berlin, Germany; 4 Department of Computer Science, Technical University Chemnitz, Germany; 5 Department of Psychology, University of Göttingen, Germany; 6 Department of Neurology, University of Lübeck, Germany; 7 Department of Psychology, Free University of Berlin, Germany

**Keywords:** consciousness, visual awareness, unconscious cognition, subliminal perception, attention

## Abstract

Psychological and neuroscience approaches have promoted much progress in elucidating the cognitive and neural mechanisms that underlie phenomenal visual awareness during the last decades. In this article, we provide an overview of the latest research investigating important phenomena in conscious and unconscious vision. We identify general principles to characterize conscious and unconscious visual perception, which may serve as important building blocks for a unified model to explain the plethora of findings. We argue that in particular the integration of principles from both conscious and unconscious vision is advantageous and provides critical constraints for developing adequate theoretical models. Based on the principles identified in our review, we outline essential components of a unified model of conscious and unconscious visual perception. We propose that *awareness* refers to consolidated visual representations, which are accessible to the entire brain and therefore globally available. However, *visual awareness* not only depends on consolidation within the visual system, but is additionally the result of a post-sensory gating process, which is mediated by higher-level cognitive control mechanisms. We further propose that amplification of visual representations by attentional sensitization is not exclusive to the domain of conscious perception, but also applies to visual stimuli, which remain unconscious. Conscious and unconscious processing modes are highly interdependent with influences in both directions. We therefore argue that exactly this interdependence renders a unified model of conscious and unconscious visual perception valuable. Computational modeling jointly with focused experimental research could lead to a better understanding of the plethora of empirical phenomena in consciousness research.

## Plethora of phenomena and paradigms in experimental consciousness research

Elucidating human consciousness remains one of the greatest and most exciting scientific challenges in the 21^st^ century. Until the 19^th^century, due to the privacy of consciousness phenomena, consciousness has been assumed to be inaccessible to empirical research and remained largely the domain of philosophy. In particular, the scientific explanation of *phenomenal awareness* (or *phenomenal consciousness* after Block, 1995), that is, the experiential qualities of sensations, has been considered as the “hard problem” of consciousness research (Chalmers, 1995; Nagel, 1974). It has been questioned whether phenomenal awareness, which is essentially defined by subjective experience, can be studied in an objective empirical manner.

Advances in experimental psychology in the 19^th^and 20^th^century and in the cognitive neurosciences at the end of the 20^th^century have rendered phenomenal consciousness accessible to empirical investigations. Since the mid-nineties of the 20^th^century, consciousness research has become a recognized area within psychology and the neurosciences, despite all epistemological problems. In particular, rigorous psychophysical work on visual masking (Bachmann, 1994; Breitmeyer & Ganz, 1976; Bridgeman, 1971) and on subliminal vi-suomotor priming as well as neurobiological studies on the neural correlates of consciousness (Crick & Koch, 1990; Singer, 1999) have convincingly demonstrated that an empirically informed approach to consciousness is possible. These advances have been made possible by focusing research on specific aspects of consciousness, such as the dissociation between conscious and unconscious perception, altered states of awareness, and amnesia. Psychological and neuroscience approaches in combination promote further progress in elucidating the cognitive and neural mechanisms that underlie phenomenal awareness and its functions in information processing.

Research in the domain of visual perception has been particularly successful because the presentation of visual stimuli can be controlled precisely with current technology. Therefore, visual perception is ideally suited for investigating the dynamics of the processes ranging from the effects of unconscious stimuli to the generation of conscious perception. Within this field, there are several lines of research. One line focuses on the neuro-cognitive mechanisms underlying the effects of unconscious visual stimuli (subliminal perception). Within this area, the focus is on the conditions for unconsciously perceived stimuli to influence (prime), that is, facilitate or inhibit, information processing and motor actions. Theories of conscious vision are informed indirectly by the limitations and potentials of unconscious vision. As various methods of rendering stimuli invisible may exert their suppressive effects at different levels of visual processing, the faculties of unconscious processing may critically depend on the precise way of eliminating conscious awareness. Research in this field helps to elucidate the chain of processes giving rise to consciousness by comparing the faculties of visual processing across different unconscious states and by identifying the specific faculties that can only by achieved in a conscious state. Those types of processes that can only be performed in a conscious state are top candidates for understanding the functions of consciousness.

In this domain, a variety of experimental paradigms has been developed for studying the effects of unconscious primes on the processing of subsequent visible target stimuli. Prime stimuli can be rendered invisible (subliminal) by masking stimuli which precede or follow the prime (for methods assessing prime visibility, see Khalid, König, & Ansorge, 2011; Schmidt, 2007). This procedure is called *masked priming*.

Here, a first observation is the remarkably rich range of processing faculties that can operate without conscious vision. Different forms of priming can be distinguished by the relation between prime and target realized in the experiment. In *response priming* (Neumann & Klotz, 1994), prime and target indicate either the same (e.g., right-hand response) or a different motor response. A second finding demonstrating the power of unconscious processing concerns the flexibility of priming. Stimuli are arbitrarily associated with the motor response and do not show any other meaningful relation (for an overview, see Schmidt, Haberkamp, & Schmidt, 2011). For instance, geometrical objects are used as primes and targets, which are assigned to alternative responses. Thereby, prime-target pairings may be congruent or incongruent in terms of their assigned response alternatives. Participants have typically to decide whether the target stimulus requires a right-hand or a left-hand response. Response priming, that is, faster responses to targets when the prime indicates the same rather than a different response, arises from automatic visuomotor response preparation triggered by the unconsciously perceived masked prime (Dehaene, Naccache, et al., 1998; Klotz & Neumann, 1999; Mattler, 2003; Neumann & Klotz, 1994; Schmidt, 2002; Verleger, Jakowski, Aydemir, van der Lubbe, & Groen, 2004; Vorberg, Mattler, Heinecke, Schmidt, & Schwarzbach, 2003). Extending the response priming paradigm it has been demonstrated that subliminal stimuli can also modulate exogenous shifts of spatial attention (Ansorge, Heumann, & Scharlau, 2002; Scharlau & Ansorge, 2003), endogenous shifts of modality-specific attention (Mattler, 2003, 2005) as well as task-specific control operations (Mattler, 2003, 2005, 2006). Much as with motor priming, attentional effects can be triggered by primes that are just voluntarily and flexibly coupled to the task at hand, for example, to attend to red and ignore green figures (see Ansorge, Horstmann, & Scharlau, 2011).

In *semantic priming* (Neely, 1991), primes and targets are meaningfully related words (or pictures) in one condition (e.g., *table-chair*) and unrelated words (or pictures) in the other condition (e.g., *car-hen*). In contrast to the response priming paradigm, primes in the congruent and incongruent conditions always afford the same response in the target task thereby ruling out any response congruency effects. Nevertheless, responses to targets that have been preceded by a semantically related prime are performed more quickly than responses to targets paired with unrelated primes (Carr & Dagenbach, 1990; Kiefer, 2002; Kiefer & Brendel, 2006; Kiefer & Spitzer, 2000). These masked semantic priming effects reflect unconscious access to the meaning of the prime which automatically pre-activates the semantic representation of the target. In addition to these pure forms of response and semantic priming paradigms, mixed paradigms are possible in which primes and targets differ with regard to both semantic relatedness and response congruency (e.g., Damian, 2001).

Findings from the different masked priming paradigms have established subliminal priming effects as a reliable and valid index of unconscious stimulus processing. However, the issue is still unresolved whether the same functional and neural mechanisms underlie the different forms of subliminal priming. Hence, although the existence of unconscious stimulus processing has been demonstrated beyond doubt in several studies, the specific mechanisms of these different forms of unconscious perception have still to be determined (Dehaene, Changeux, Naccache, Sackur, & Sergent, 2006).

A complementary line of consciousness research focuses on the functional and neural mechanisms underlying the generation of conscious percepts. This research aims at elucidating the interface between conscious and unconscious perception by employing a variety of experimental paradigms. For instance, the neural correlates of perceptual phenomena have been studied with the help of multistable stimulus configurations that give rise to alternative interpretations. Examples are *unstable visual percepts* during binocular rivalry or in ambiguous fi-gures (Engel, Fries, König, Brecht, & Singer, 1999; Haynes, Deichmann, & Rees, 2005; Leopold & Logothetis, 1996; Mendola, Dale, Fischl, Liu, & Tootell, 1999). Furthermore, experimentally perturbing percepts has been used as a technique for inferring the conditions needed to generate a complete conscious percept (e.g., Mattler & Fendrich, 2010). Most paradigms are designed to investigate experimental factors which reduce conscious stimulus identification either due to masking, interfering stimuli, or attentional distraction. While visual masking presumably prevents the consolidation of the percept within the visual system (Breitmeyer & Ömen, 2006; Haynes, Driver, & Rees, 2005), the influence of a central top-down gating mechanism is postulated as the relevant factor for experimentally *distractor-induced blindness*. Such blindness has been exemplified in detecting coherent motion when preceded by to-be-ignored distractor motion (Niedeggen, Hesselmann, Sahraie, Milders, & Blakemore, 2004). Finally, reduced attentional resources are assumed to play a major role for the emergence of consciousness as indicated by the *attentional blink* phenomenon (Shapiro, Arnell, & Raymond, 1997; Verleger et al., 2009). To elicit the attentional blink, two targets are embedded in a series of rapidly displayed visual stimuli. The second target may not be consciously reportable if the first target is actively processed and the temporal interval between both targets ranges between 200-500 ms. Is this due to the fact that attention is a necessary (but not sufficient) requirement for consciousness but blocked by the first target so that is momentarily not available for the second target in the sequence?

One can see from these experimental paradigms that they have the potential to provide insight into the mechanisms at a functional level, which contribute to the selection and integration of the visual information constituting the conscious percept. At a neural level, activity in brain areas has been identified which correlates with conscious percepts.

In order to successfully elucidate the mechanisms of conscious and unconscious visual perception both at a functional behavioral and at a neural level, the processes behind the phenomena must be identified by a broad range of methods. Behavioral measures provide an insight in the functional properties of the neuro-cognitive system. As they are the output of the entire processing chain, they cannot be used for on-line monitoring of cognitive processing. However, given the appropriate experimental manipulations, a particular behavioral measure may tap into specific parts of the neuro-cognitive system (for an example, see Breitmeyer, Koc, Ziegler, & Ömen, 2008).

Neurophysiological measures, in contrast, convey information on the functional neuroanatomical architecture of the cognitive system (functional magnetic resonance imaging, fMRI) and on the temporal and spatial time course of processing (event-related potentials, ERPs; magnetoencephalography, MEG). Important complementary methods to these are studies with brain-damaged patients and with transcranial magnetic stimulation (TMS). Unlike neurophysiological measures, which only provide correlational evidence, TMS and patients studies allow to determine whether activity in some specific brain area is necessarily involved in a given cognitive process, such as those giving rise to consciousness. If a behavioral effect disappears because of damage to a particular brain area or its transient modulation by TMS, this finding suggests that this area necessarily plays a functional role for producing this effect and is part of the underlying processes.

This brief overview of research in the past decades shows that previous studies pragmatically investigated certain aspects of consciousness phenomena by heterogeneous experimental paradigms. The use of various experimental approaches and the focus on different phenomena renders it difficult to bridge the gaps between the different research areas and to develop a unified theory of conscious and unconscious visual perception.

## Identifying general principles underlying conscious and unconscious visual perception

The diversity of consciousness research renders it difficult to promote the integration of separate lines of research on consciousness and visual awareness. Nevertheless, the findings of the seemingly heterogeneous studies in consciousness research provide a rich source of evidence to identify general principles that play a central role in conscious and unconscious visual perception. Some of them might be common to several phenomena or experimental paradigms; others might apply to one area only. In this section, we will describe general principles, by addressing five major questions which may help to systematize the plethora of findings in research on visual awareness. Due to the vast extent of the field, this review necessarily has to focus on a selection of phenomena and of experimental paradigms.

### Which cognitive systems and/or brain regions are relevant for generating conscious visual percepts?

According to one currently popular view, consciousness in general and visual awareness in particular depends on coordinated processing in several cognitive systems and correspondingly involves a large network of brain areas (Dehaene & Naccache, 2001; Haynes, Driver, & Rees, 2005). However, although a coherent, unitary percept is one of the key features of conscious experience at a subjective level, at a neural level this unitary percept may actually arise from distributed processing in lower-and higher-order visual areas of the occipito-temporal cortex (Lamme, 2003) as well as in attentional areas outside visual cortex (Hamker, 2007). Therefore, consciousness is unlikely to be the simple result of processing in a single “consciousness” module (Dennett & Kinsbourne, 1992): Besides the various components of the visual system (primary, secondary, and visual association cortex), prefrontal and temporo-parietal brain areas involved in attention and cognitive control (Posner & Driver, 1992) are crucial in the generation of visual awareness that can be expressed in verbal reports (Hulme, Friston, & Zeki, 2009; Lamme, 2003). The modularity of visual information processing raises the question regarding the role of the different modules in the generation of conscious experience. According to one view, there are several states of micro-consciousness and the experience of the unity of consciousness is an illusion (Zeki, 2003).

Despite the distributed nature of processing subserving visual awareness, the question arises as to whether some brain areas or cognitive systems are more important than others. Damage to primary visual cortex in the occipital lobe results in cortical blindness although visual abilities of these patients can be improved by perceptual learning induced by repeated stimulation of the impaired visual field (Trevethan, Urquhart, Ward, Gentleman, & Sahraie, 2012): Besides visual areas, brain regions supporting attention appear to play a crucial role in visual awareness. In particular, right temporo-parietal areas that are damaged in patients with neglect syndrome are crucial in generating a conscious visual percept, presumably by guiding attention in space (Karnath, Ferber, & Himmelbach, 2001; Karnath, Fruhmann-Berger, Kuker, & Rorden, 2004). Based upon this and similar findings, it has been suggested that visual processing in the right hemisphere is dominating the conscious percept. Using two streams with rapid serial visual presentation in each hemifield, the right hemisphere has been shown to be superior in conscious visual perception of the second of two targets embedded in these streams (migasiewicz et al., 2010; Verleger et al., 2009). This right-hemisphere (RH)/ left-visual-field (LVF) advantage proved robust across cultures (migasiewicz et al., 2010) and against interference by repetitive TMS (Verleger et al., 2010). It is suggested that the RH/LVF advantage in visual perception reflects an attentional bias under high attentional load, presumably due to the superiority of the RH attentional system for guiding attention in space.

### Which mechanisms are responsible for the selection and integration of visual information that contributes to the conscious visual percept?

 It is well documented that only a fraction of the physical information that reaches the retina contributes to the conscious visual percept. Stimuli that would be visible if presented in isolation are invisible when subsequently masked by a spatially overlapping visual pattern or by a surrounding metacontrast stimulus (Breitmeyer & Ömen, 2006). Although there are several competing models on visual masking, they all converge on the assumption that awareness requires some consolidation of information within the visual system depending on interactions within visual areas (Bachmann, 2007; Breitmeyer, 2007; Bridgeman, 2007; Enns & Di Lollo, 2000; Macknik & Martinez-Conde, 2007; Scharlau, Ansorge, & Breitmeyer, 2006). In addition, phenomena such as inattentional blindness (Mack & Rock, 1998; Rees, Russell, Frith, & Driver, 1999), change blindness (Rensink, O‘Regan, & Clark, 1997, and the attentional blink (Shapiro et al., 1997) show that attentional top-down amplification of the target representation contributes to a successful consolidation process leading to visual awareness (Kessler et al., 2005). The N2pc component of the ERP reflects the allocation of visual attention to potentially task-relevant stimuli. By analyzing the detailed time-course of N2pc, Verleger, urawska vel Grajewska, and Jakowski (2012) provide evidence for the oscillatory nature of the generating process, possibly reflecting recurrent loops of processing in visual cortex. 

 A neural instance of the global workspace theory assumes that stimuli require a sufficiently strong activation level to enter the global workspace, while stimuli with a slightly weaker activation level quickly decay (Dehaene et al., 2006). Dehaene and colleagues (2006) distinguish accessibility from access to account for conflicting neuroimaging data. A weak or interrupted stimulus activates only early visual areas. Its subliminal processing can be influenced by the subject’s attention using an attentional set which is already prepared prior to the task (Kiefer & Martens, 2010). A sufficiently strong stimulus is processed preconsciously but temporarily buffered in a non-conscious store because of a lack of top-down attentional amplification (Dehaene et al., 2006) as in attentional blink or inattentional blindness paradigms or because of a failure to encode the stimulus in working memory circuits as in the distractor-induced blindness paradigm. Once attentional resources are available and the central workspace is freed, a pre-conscious stimulus might ultimately achieve conscious access which is manifested by intense activation spreading from visual areas to the fronto-parietal attentional network. Presumably, this information exchange between visual areas and the fronto-parietal attentional network is achieved by coordinated oscillatory activity in different frequency bands across multiple brain regions as suggested by electrophysiological recordings during the attentional blink (Janson & Kranczioch, 2011). 

However, the neural structures that mediate the global workspace are less clear. While prefrontal cortex subserving attention and working memory functions is certainly a substantial part of the global workspace, its understanding will also help to reveal the transition from pre-conscious to conscious perception. Among others, the basal ganglia have been proposed to play an important role in working memory control (Brown, Schneider, & Lidsky, 1997; Middleton & Strick, 2000) as supported by recent computational models of basal ganglia function (O’Reilly & Frank, 2006; Vitay & Hamker, 2010).

Studies on distractor-induced blindness suggest that awareness not only depends on attentional amplification through global workspace circuits, but also on a post-sensory central gating mechanism presumably exerted by prefrontal circuits that permits or prevents stimuli to enter conscious awareness (Niedeggen et al., 2004). In the paradigm of distractor-induced blindness (Sahraie, Milders, & Niedeggen, 2001), conscious access to simple visual features (i.e., motion or orientation) is impaired during rapid serial visual presentation when the same feature had to be ignored as a distractor previously. Psychophysical and electrophysiological experiments on this effect indicated that distractor stimuli which share visual features with an upcoming target will activate an inhibitory mechanism. The strength of the inhibition is primarily defined by the number of distractors presented (Hesselmann, Niedeggen, Sahraie, & Milders, 2006). ERP studies indicated that the inhibition tags a central process and does not directly affect sensory processing (Niedeggen et al., 2004) because ERP correlates of the sensory processing of visual motion (Niedeggen, Sahraie, Hesselmann, Milders, & Blakemore, 2002), or orientation changes did not differ between detected and missed events. These results are inconsistent with suggestions that the conscious representation of visual stimuli is closely related to activity in the occipital cortex (Pins & Ffytche, 2003). Together with similar findings reported in studies on the attentional blink (Dehaene, Sergent, & Changeux, 2003; Luck, Vogel, & Shapiro, 1996), the data on distractor-induced blindness suggest that activation of visual cortex is necessary, but not sufficient, to generate visual awareness. Although the precise nature of the central gating mechanisms has to be elucidated further, these results also indicate that encoding of stimuli into working memory circuits, which is mediated by higher-level control mechanisms (Zhang, Zhou, & Martens, 2009) is necessary to give rise to visual awareness in addition to sensory activation. Presumably, currently active or inhibited attentional task sets are the basis of this gating mechanism and determine, which information is encoded into working memory (Michael, Kiefer & Niedeggen, 2012).

### What is the role of top-down influences for conscious and unconscious perception?

As noted above, top-down attention leads to amplification of the sensory representation of a target stimulus (Hamker, 2005, 2007), an important prerequisite for visual awareness (Dehaene et al., 2006). Attentional amplification is achieved by top-down signals from prefrontal cortex (Haynes, et al., 2007) that modulate activity of single neurons in sensory brain areas in the absence of any sensory stimulation (Tomita, Ohbayashi, Nakahara, Hasegawa, & Miyashita, 1999) and significantly increase baseline activity in the corresponding target region (Reynolds, Chelazzi, & Desimone, 1999). Electrophysiological evidence in behaving animals suggests that such attentional sensitization is instantiated and sustained through selective neuronal synchronization of rhythmic activity at fast and slow temporal scales within and between neuronal groups (Womelsdorf & Fries, 2007).

Neuroimaging studies in human participants showed that perceiving a cue that indicates what will be the relevant dimension of the target is associated first with increased activity in prefrontal areas (Bode & Haynes, 2008; Hopfinger, Buonocore, & Mangun, 2000; Hopfinger, Woldorff, Fletcher, & Mangun, 2001). Second, in posterior brain areas, the target region of attentional control, attention to some specific stimulus dimension increases the level of baseline activity in the corresponding sensory region, even when visual stimulation is kept constant (Martinez-Trujillo & Treue, 2004; Serences, Saproo, Scolari, Ho, & Muftuler, 2009; Song, Rowland, McPeek, & Wade, 2011).

 Similar to phenomena in conscious perception, unconscious processes are susceptible to attentional control. Unconscious priming has been shown to depend on attentional top-down amplification. Priming was only obtained when the masked prime was presented within the time window of attention (Kiefer & Brendel, 2006; Naccache, Blandin, & Dehaene, 2002). Furthermore, top-down control processes can constrain processing of unconsciously perceived stimuli if they misguide overt behavior (Jakowski, Skalska, & Verleger, 2003; Wolbers et al., 2006). Presumably, top-down control is reactively engaged in response to previous consciously perceived errors in order to suppress interfering subliminal information. The influence of attention on unconscious visual processing is even more specific because masked response priming has been shown to depend on action intentions and task sets: Unconsciously perceived masked primes trigger responses only if they are congruent with the current intentions of a person (Ansorge et al., 2002; Ansorge & Neumann, 2005) and represent possible release conditions for prepared actions (Eckstein & Perrig, 2007; Kiesel, Kunde, & Hoffmann, 2007; Kiesel, Kunde, Pohl, Berner, & Hoffmann, 2009; Kunde, Kiesel, & Hoffmann, 2003). Furthermore, Ng, Chan, and Schlaghecken (2012) showed that unconscious visuo-motor processes are differentially influenced by cognitive control settings induced by specific emotional states (subclinical depression vs. anxiety). Using a novel procedure for masked priming of words and of geometrical shapes, attentional task sets were shown to influence unconscious semantic and visuo-motor processes selectively (Kiefer & Martens, 2010; Martens & Kiefer, 2009). These results demonstrate that preemptive top-down control of unconscious processes coordinates the perceptual and semantic processing streams: An attentional sensitization mechanism enhances or attenuates the responsiveness of semantic and visuo-motor processing pathways to incoming subliminal stimuli depending on the currently activated task set (Kiefer, 2007; Kiefer & Martens, 2010), thereby differentially influencing subsequent subliminal semantic and visuo-motor priming. This attentional mechanism optimizes ongoing processing toward the pursuit of an intended goal and therefore ensures the adaptability of cognition even in the unconscious domain (see also Kiefer, Adams, & Zovko, 2012).

 In a continuation of this line of research, the capture of visuo-spatial attention by unconscious stimuli likewise was shown to depend on the match between these stimulus features and a fitting top-down search template directed towards the relevant visual features of the targets (Ansorge, Horstmann, & Worschech, 2010; Ansorge, Kiss, & Eimer, 2009; Held, Ansorge, & Müller, 2010). With respect to the allocation of visuo-spatial attention, it was concluded that the feedforward phase of visual processing was entirely determined by top-down task sets (Ansorge, Horstmann, & Scharlau, 2010). Top-down effects on attentional capture by unconscious stimuli are discussed in detail by Ansorge, Horstmann, and Scharlau (2011) and by Reuss, Pohl, Kiesel, and Kunde (2011) . 

The influence of attention on unconscious processing also demonstrates that attention and consciousness are distinct mental pheno-mena (cf. Mack & Rock, 1998). Some researches even argue against a significant role of attention in determining the content of awareness (Van Boxtel, Tsuchiya, & Koch, 2010). However, neither *attention* nor *consciousness* are well defined. A unified model of conscious and unconscious visual perception must refer to unambiguously clear definitions of such terms preferably manifested by computational models (for a recent proposal, see Trapp, Schroll, & Hamker, 2012).

### Are there common mechanisms of all types of unconscious visual processing (perceptual, motor, and cognitive)?

Subliminal priming studies show that unconscious visual stimuli can trigger processes at perceptual, motor, and semantic levels but also at levels of cognitive control. For instance, masked stimuli can elicit perceptual (Scharlau & Ansorge, 2003; Scharlau & Horstmann, 2006), visuo-motor (Dehaene, Naccache, et al., 1998; Neumann & Klotz, 1994; Vorberg et al., 2003), semantic (Carr & Dagenbach, 1990; Kiefer, 2002), attentional (Ansorge et al., 2002; Mattler, 2003, 2005), and control-related effects (Mattler, 2003, 2005, 2006) on the processing of subsequently presented visible targets. Although involving distinct processing streams in the brain, these different subliminal priming effects seem to be governed by fairly similar mechanisms: By their dependence on attention, intention and task sets, all of these types of processes triggered by the unconscious primes are susceptible to top-down control. As already discussed in detail above, unconscious priming, both visuo-motor and semantic priming, has been shown to require attentional top-down amplification. Subliminal priming effects were only obtained when the primes were attended to (Kiefer & Brendel, 2006; Naccache et al., 2002) or when attentional resources were available (Martens & Kiefer, 2009). Furthermore, masked visuo-motor priming and semantic priming have been shown to depend on action intentions (Ansorge et al., 2002; Ansorge & Neumann, 2005; Eckstein & Perrig, 2007; Kiesel et al., 2007, 2009; Kunde et al., 2003) and task sets (Kiefer & Martens, 2010; Martens, Ansorge, & Kiefer 2011; Martens & Kiefer, 2009).

 Beyond these general preconditions for priming effects of unconscious stimuli, evidence has accumulated which suggests that at least part of these effects of masked stimuli result from a common mechanism of information integration. Vorberg et al. (2003) described this simple integration mechanism which accounts for the temporal dynamics of priming effects on performance measures in forced-choice reaction time tasks. This model has been specified to account for visuo-motor priming effects on reaction times and error rates. However, when the dynamics of non-motor priming effects has been examined, findings suggest a comparable time course for priming of attention as well as of priming of cognitive control operations (Mattler, 2003, 2005). Therefore, the mechanism of information integration might provide a general framework to account for subliminal priming effects that are dissociated from mechanisms which generate conscious percepts of the corresponding stimuli (Mattler, 2003, 2005; Schmidt & Vorberg, 2006). 

### How does conscious visual processing differ from unconscious visual processing?

The review above shows that conscious and unconscious visual processing share a variety of principles: Priming experiments indicate that visual processing is comparable for conscious and unconscious viewing conditions within the first hundred milliseconds of stimulus processing (Kiefer & Spitzer, 2000; Vorberg et al., 2003; Vorberg, Mattler, Heinecke, Schmidt, & Schwarzbach, 2004). In line with this, unconscious visual words can elicit motor activation effects based on a word’s long-term meaning (e.g., Ansorge, Kiefer, Khalid, Grassl, & König, 2010), much as it has been shown with conscious words (Proctor & Vu, 2002). Furthermore, conscious and unconscious visual processing are similarly susceptible to attentional control and depend on attentional resources.

 However, there are also important differences: Unlike conscious control, top-down control of unconscious cognition requires task sets to be set up in advance of stimulus presentation (i.e., preemptive control) and cannot be initiated reactively in response to the sensory input (Ansorge, Fuchs, Khalid, & Kunde, 2011; Ansorge & Horstmann, 2007; Ansorge et al., 2009; Kiefer, 2007; Kiefer & Martens, 2010). In line with this assumption, unconsciously induced conflict does not seem to alter cognitive control settings in contrast to conscious stimuli (Merikle, Joordens, & Stolz, 1995). Furthermore, only conflict elicited by conscious, but not by unconscious stimuli leads to an adjustment of conflict regulation in a subsequent trial (Ansorge et al., 2011; Greenwald, Draine, & Abrams, 1996; Kunde, 2003). The relation between consciousness and cognitive control is further discussed by Kunde, Reuss, and Kiesel (2012) . 

A further difference between conscious and unconscious visual processing concerns the stability of processes as a function of time: For instance, under unconscious conditions, semantic priming effects decayed relatively fast (i.e., within about 200 ms) whereas under conscious conditions priming increased with time (Greenwald et al., 1996; Kiefer & Spitzer, 2000). This finding suggests that processes triggered by unconscious visual stimuli fade quite fast as a function of time, presumably because unconscious visual representations are not consolidated and are therefore temporally less stable (see also Mattler, 2005). In contrast, conscious visual representations are temporally very stable, because they are highly consolidated within the visual systems and additionally benefit from active maintenance in working memory (Dehaene et al., 2006; Merikle & Daneman, 1998). This suggestion is in line with findings from intracranial recordings in humans demonstrating increased neural activity in widespread higher-order visual areas for consciously perceived stimuli starting at about 150 ms that outlasted stimulus presentation (Fisch et al., 2009). Conscious and unconscious visual processing do not only differ in the temporal stability of visual representations, but also with regard to the speed at which different visual features are processed: At unconscious levels form processing proceeds faster than surface processing, whereas at conscious levels form processing proceeds slower than surface processing (see Breitmeyer & Tapia, 2011).

## Towards a unified model of conscious and unconscious visual perception

The general principles that we have identified above to characterize conscious and unconscious visual perception may serve as important building blocks for a unified model to explain the plethora of findings in these domains. We strongly believe that in particular the integration of principles from both conscious and unconscious vision is advantageous and provides critical constraints for developing adequate theoretical models. In the following part, based on a synthesis of these general principles, we outline essential elements, which any unified model of conscious and unconscious visual perception should incorporate according to our view. Many elements and mechanisms that we describe below are already partially realized in existing theories of cognitive control (Botvinick, Braver, Barch, Carter, & Cohen, 2001; Dehaene, Kerszberg, & Changeux, 1998; Posner & Rothbart, 1998), consciousness (Crick & Koch, 1990; Dehaene & Naccache, 2001; Lamme, 2003), attention (Hamker, 2005; Pessoa, Kastner, & Ungerleider, 2003; Zirnsak, Beuth, & Hamker, in press), and unconscious processing (Ansorge & Neumann, 2005; Kiefer & Martens, 2010; Kunde et al., 2003; Neumann, 1990). However, to achieve a breakthrough we strongly believe that the different mechanisms, which served to explain heterogeneous phenomena of conscious and unconscious vision in the past, have to be integrated within one single model. This envisioned unified model should in particular elaborate on the nature of attentional control mechanisms that influence both conscious and unconscious visual perception, preferably at a formal computational level (for a recent proposal, see e.g., Trapp et al., 2012).

In line with many other models, we assume that visual awareness depends on consolidation of representations within the visual system. Consolidation of representations is achieved by feedforward and feedback processing (re-entrant processing) within lower-level and higher-level visual areas. Successful consolidation of a visual representation is characterized by a sustained and stable neural activation pattern, which lasts for a few hundred milliseconds. In addition to physical stimulus strength (defined by duration, luminance, or contrast) and temporal distance to preceding or following stimuli (i.e., visual masks), consolidation benefits from top-down attention, which amplifies the visual representation of the target stimulus (Dehaene et al., 2006; Hamker, 2005, 2007). We assume that stimulus strength, temporal distance to other (masking) stimuli (target-mask stimulus onset asynchrony), and attentional amplification influence consolidation in a compensatory manner (for the conjoint influence of attention and target-mask SOA, see Bruchmann, Hintze, & Mota, 2011): Target stimuli of sufficient strength presented at a large temporal distance to subsequent stimuli (i.e., in the absence of masking effects) can be efficiently consolidated even when they receive no or only little attentional amplification (Van Boxtel et al., 2010). In contrast, stimuli with only little physical strength (e.g., briefly presented) followed by other stimuli in close temporal succession (during visual masking) remain unconscious although they are attended to (Ansorge et al., 2009). Hence, in line with previous proposals, we assume that attention and visual awareness are related, but distinct phenomena (Kiefer & Martens, 2010; Koch & Tsuchiya, 2007; Lamme, 2003): *Attention* refers to an amplification of stimulus representation in the task-relevant processing pathways through a prefrontal top-down signal, which increases the probability that a neuron or a population for neurons fires at a given activation level (Trapp et al., 2012). Attentional amplification is not uniformly distributed across both visual fields, but exhibits are bias towards the left visual field: Attentional amplification is more pronounced in the left visual field particularly under high attentional load, for instance for visual identification in rapid serial visual presentation or for complex visual search. This RH/LVF attentional bias is presumably due to the superiority of right temporo-parietal areas for guiding attention in space when stimuli in both visual fields compete for attentional amplification in rapid succession.

According to our view, attention facilitates the visual consolidation process, but is neither necessary nor sufficient for visual awareness. *Awareness* refers to consolidated visual representations, which are accessible to the entire brain and therefore globally available. Visual awareness not only depends on attention-mediated consolidation within the visual system, but is additionally the result of a post-sensory gating process (Niedeggen et al., 2004), which determines whether consolidated stimuli are encoded into working memory in accordance with active task sets. Consolidated stimuli only reach awareness and are available for verbal report, when they are encoded in working memory circuits. Our suggestion of a post-sensory gating process into working memory circuits is derived from studies on distractor-induced blindness where the presentation of distractors led to deficits in visual awareness while activity in the sensory system remained unaffected.

We therefore propose that visual awareness depends on both consolidation within the visual system and subsequent post-sensory encoding into working memory. The latter is accomplished by a central gating mechanism according to currently active task sets. If one of the processes fails, the visual stimulus remains unconscious because consolidation is assumed to be a prerequisite for working memory encoding.

 As far as the involved brain areas are concerned, consciousness likely comprises modality-specific association cortices to represent the content of consciousness and those areas that dynamically bind cortical activity into a single consciously perceived state. The binding mechanism must be an active process that dynamically links particular brain areas together. An outline of a theoretical concept is given by Trapp et al. (2012) . Prefrontal cortex and basal ganglia are involved in cognitive control and working memory. However, they appear also to be the core structures determining the content of consciousness. In addition, the thalamus appears to implement the binding itself by rapidly switching transmission in cortical areas and thus allowing a dynamic reconfiguration of functional connectivity. 

Even if a stimulus does not give rise to a conscious percept, it can trigger processes in various brain systems and thereby influence cognition and behavior as shown by subliminal priming studies: Unconscious stimuli elicit processes not only in systems functionally and anatomically closely related to the visual system such as visuo-motor, semantic, and affective brain circuits, but also in higher-level systems devoted to attention and cognitive control: Unconscious visual stimuli even modulate the focus of attention and activate specific task sets demonstrating their far reaching influence on top-down control. Unconscious processing in the different higher- and lower level systems seems to be based on similar information integration processes as shown by the temporal dynamics of subliminal priming.

Most notably, unconscious processing is not autonomous and invariantly triggered whenever a subliminal stimulus is presented, but crucially requires an appropriate configuration of the cognitive system by attention, task sets, and action intentions in advance of stimulus presentation. We assume that unconscious stimulus processing, similar to conscious processing, can be sensitized by attention. This attentional sensitization mechanism is thought to be driven by a prefrontal top-down signal and enhances unconscious processing in task-relevant pathways (e.g., visuo-motor, semantic, affective, attentional, etc.) while it attenuates processing in task-irrelevant pathways. Hence, unconsciously perceived stimuli can trigger only those processes that match currently active attentional task sets and action goals. In line with our suggestion, top-down attentional modulation of unconscious processing is empirically well documented. The fact that attentional influences affect unconscious processing and are not restricted to conscious perception is another argument for our proposal that attention and consciousness cannot be equated (Lamme, 2003).

Given that unconscious and conscious visual processing is assumed to differ only with regard to visual consolidation and/or post-sensory gating into working memory, it immediately follows that both visual processing modes exhibit many similarities in their potential to trigger processes in other cognitive systems and in their susceptibility to attentional control.

However, as unconscious visual perception lacks visual consolidation and/or encoding into working memory circuits, several functional differences emerge between unconscious and conscious visual processing modes. First, conscious visual representations are temporally more stable than unconscious representations because they are actively maintained in working memory circuits. Second, top-down control for conscious processing is certainly more flexible than for unconscious processing. Preemptive control can be exerted for both conscious and unconscious stimulus presentation, whereas only consciously perceived stimuli are susceptible to reactive control in response to ongoing or completed stimulus processing. Reactive control is presumably restricted to conscious visual processing because this control mode requires monitoring the consequences of stimulus processing in working memory. As unconscious stimuli do not leave traces in working memory circuits, top-down control of unconscious cognition must occur implicitly on the grounds of currently activated action goals or the outcome of overt behavior. As a consequence, intentional application of control and online modification is restricted to conscious vision. Thus, conscious stimulus processing allows for a greater adaptability and flexibility of top-down control than processing under unconscious viewing conditions.

## Conclusion

Based on a literature review of the plethora of empirical phenomena in consciousness research, we have identified important principles underlying conscious and unconscious visual perception and outlined essential elements of a unified model. We propose that conscious visual perception depends on a consolidation process within the visual system and a subsequent post-sensory gating process, which determines, whether a stimulus is encoded in prefrontal working memory circuits. Only if both processes are successfully completed, the stimulus is consciously perceived and reportable; otherwise the stimulus remains unconscious. Attention facilitates the consolidation process by amplifying the visual stimulus representation and therefore increases the likelihood for generating a conscious percept, but is neither necessary nor sufficient for visual awareness.

We further propose that amplification of visual representations by attentional sensitization is not exclusive to the domain of conscious perception, but also applies to visual stimuli, which remain unconscious. Hence, processing of both consciously and unconsciously perceived visual stimuli depends on top-down attention. Given the appropriate attentional state, set up in advance of stimulus presentation, which sensitizes the corresponding neural processing pathways, unconscious stimuli trigger processes in the visuo-motor, semantic, and affective systems and influence even cognitive control processes. Conscious and unconscious processing modes are highly interdependent with influences in both directions. We therefore argue that exactly this interdependence renders a unified model of conscious and unconscious visual perception valuable.

Although much progress has been made to elucidate the neuro-cognitive mechanisms of conscious and unconscious visual perception, we want to highlight four issues, which represent important areas of future research:

1. The nature of the proposed visual consolidation process, which is assumed to be an important factor for visual awareness, has to be further specified. Although re-entrant processing within visual areas might be important for consolidation, conclusive evidence is missing so far. Likewise, it has to be better elucidated at a functional and neural level how the prefrontal attentional top-down signal influences in interaction with temporo-parietal areas the consolidation of visual representations.

2. Although several lines of evidence suggest the importance of a putative prefrontal post-sensory gating mechanism for generating visual awareness, its precise function and neural substrate as well as its interaction with the visual consolidation process in occipito-temporal areas remains to be determined.

3. We are only at the beginning of understanding the interplay between conscious and unconscious visual processing modes: On the one hand, attentional sets appear to influence unconscious processing in congruency with higher-level action goals. On the other hand, unconsciously perceived stimuli seem to modulate conscious percepts as well as cognitive control settings. It is highly desirable to better characterize the functional and neural mechanisms underlying these mutual influences between conscious and unconscious domains.

4. The future development of a unified computational model of conscious and unconscious visual perception is certainly essential to formally specify the mechanisms that we have outlined here and to derive novel predictions to be tested in future experiments. We strongly believe that an approach that integrates research on conscious and unconscious vision is particularly suited to explain the neuro-cognitive mechanisms giving rise to human consciousness. Computational modeling jointly with focused experimental research could lead to a better understanding of the plethora of empirical phenomena in consciousness research, particularly by its potential to reveal underlying mechanisms for multiple observations and to formulate rigid testable predictions.
